# Aged‐Related Fibroblast Activation Protein Expression in Skeletal Muscles Evaluated by PET Imaging

**DOI:** 10.1002/jcsm.13730

**Published:** 2025-02-16

**Authors:** Minseok Suh, Joonhyung Gil, Yeon‐Koo Kang, Hongyoon Choi, Gi Jeong Cheon

**Affiliations:** ^1^ Departments of Nuclear Medicine, Seoul National University Hospital Seoul National University College of Medicine Seoul South Korea

**Keywords:** ageing, FAP, FAPI PET, muscle density, skeletal muscle

## Abstract

**Background:**

Fibroblast activation protein (FAP) is prominently involved in the tumour microenvironment and tissue remodelling processes in most cancers, and its expression is also noted in normal skeletal muscle. This study aims to explore the relationship between FAP expression and age‐related muscle characteristics through FAP inhibitor (FAPI) PET/CT imaging.

**Methods:**

This retrospective analysis studied 54 patients with lung cancer (*n* = 27) and pancreatic cancer (*n* = 27) using FAPI PET/CT. Imaging‐based muscle features including the mean standardised uptake value (SUVmean), skeletal muscle index (SMI) and Hounsfield units (HU) were evaluated. Age‐related FAP expression in skeletal muscles was also evaluated using the Genotype‐Tissue Expression (GTEx) dataset. Statistical analyses included Spearman's rank correlation and Kruskal–Wallis test, with a *p*‐value of less than 0.05 considered significant.

**Results:**

Analysis revealed a moderate to strong positive correlation between FAPI SUVmean and age (*ρ* = 0.368, *p* = 0.006), with older age groups showing higher muscle uptake. Within specific cohorts, the FAPI‐74 group demonstrated a stronger correlation (*ρ* = 0.500, *p* = 0.008) compared to the FAPI‐46 group (*ρ* = 0.319, *p* = 0.105). SUVmean also correlated negatively with muscle density (HU) (*ρ* = −0.298, *p* = 0.029), suggesting an association with higher fat infiltration. GTEx data supported these findings, showing a significant increase in FAP expression across age groups (*p* < 0.001), with the highest median FAP in the 70–79 age group.

**Conclusions:**

This study demonstrates an age‐related increase in FAPI uptake in skeletal muscle, correlated with changes in muscle density and fat infiltration. The role of FAP extends beyond pathology to normal muscle, indicating broader biological functions. Accordingly, FAPI PET shows promise for assessing age‐related muscle health and quality.

## 1 Introduction

Fibroblast activation protein (FAP) is predominantly expressed in cancer‐associated fibroblasts within the tumour stroma of 80%–90% of cancers, playing a pivotal role in modulating tumour behaviour [1]. It promotes angiogenesis, migration and proliferation, thereby influencing the progression and spread of cancer [2]. The development of FAP inhibitors (FAPIs), especially those containing DOTA coupled with ^68^Ga for positron emission tomography (PET) imaging, has emerged as a promising advance in oncologic imaging [3, 4]. FAPI PET/computed tomography (CT) imaging is distinguished by its superior tumour‐to‐background contrast, rapid tracer kinetics and minimal background uptake in critical organs [5, 6]. While FAP overexpression is prominently visible in areas of tissue remodelling, such as in degenerative lesions or throughout the wound healing process, it is well‐documented that degeneration can show noticeable FAP activity [7]. However, it is intriguing to observe that even normal skeletal muscle frequently demonstrates prominent FAPI uptake. This phenomenon suggests that FAP expression, while typically associated with pathological conditions, can also be a feature of uninjured muscle, indicating a broader spectrum of biological activity than previously understood. However, the specific roles and clinical implications of FAP expression in the skeletal muscle, particularly its association with various clinical factors, remain largely unexplored. In this study, we aim to elucidate the relationship between FAP expression, age and muscle quality.

## 2 Materials and Methods

### 2.1 Study Participants

Our retrospective study is based on two prospective cohorts from a single‐centre, with the study designs and participant selection criteria tailored to explore specific clinical inquiries. The first cohort (*n* = 27) focused on patients with nonsmall cell lung cancer (NSCLC) planning for curative surgery. The second cohort (*n* = 27) targeted individuals with newly diagnosed pancreatic ductal adenocarcinoma (PDAC). The study adhered to the ethical standards outlined in the Helsinki Declaration, and approval was obtained from the Institutional Review Board (H‐2101‐064‐1188 & H‐2112‐110‐1284).

### 2.2 Image Acquisition and Analysis

We utilised image data acquired 60‐min postinjection from the cohorts. The first cohort, consisting of NSCLC patients, underwent PET/CT scans using [^68^Ga]FAPI‐46, while the second cohort of PDAC patients received [^18^F]F‐FAPI‐74. For both cohorts, imaging was performed using Biograph mCT 40 or 64 scanners (Siemens Healthineers, Erlangen, Germany). CT images were acquired using the following parameters: 120 kVp, 35 mAs, slice thickness 5 mm, slice increment 3 mm and pitch 1.2.

Image analysis was performed using MIM software (MIM Software Inc., Cleveland, OH, USA). Muscle segmentation was performed at the L3 lumbar spine level. Initially, a rough region of interest (ROI) was manually outlined on axial CT slices to include visible muscle groups. Segmentation was refined by applying Hounsfield Unit (HU) thresholds in two steps: first, voxels with HU values ≥ − 29 were selected to define an initial muscle region, and next, voxels with HU values ≥ 150 were subtracted from this region to exclude high‐density structures such as bone. The resulting segmented area, containing voxels within the −29 to 150 HU range, was defined as skeletal muscle tissue (Figure S1). From these delineated areas, the following features were extracted: the mean standardised uptake value (SUVmean) of FAPI, the skeletal muscle index (SMI) (cm^2^/m^2^) and the average HU. The SMI was defined as the skeletal muscle cross‐sectional area (cm^2^) divided by the square of the patient height (m^2^).

### 2.3 Age‐Associated Gene Expression

RNA‐seq data for nondiseased muscle tissue from adult individuals were obtained from the Genotype‐Tissue Expression (GTEx) project, an open database containing a wide range of human tissue samples [8]. To process the data, recount3 pipeline was used [9] (https://rna.recount.bio/), a widely used tool for large‐scale RNA‐seq data analysis. For querying normal muscle tissue data with associated age information, we employed R software (version 4.1.1, https://www.r‐project.org/) in conjunction with the recount3 package. The data were filtered by subsetting the ‘MUSCLE’ dataset from the Genotype‐Tissue Expression (GTEx) project. This selection yielded a final dataset consisting of RNA‐seq data from 803 muscle tissue samples, each associated with corresponding demographic and clinical metadata, including age. Transcript counts of FAP were normalised to counts per million (CPM) for each sample using the cpm function from the edgeR package. To compare FAP expression across different age groups, log2 (cpm) values were calculated and analysed according to age.

### 2.4 Statistics

All statistical analyses were performed using R software (version 4.1.1), with a *p*‐value of less than 0.05 considered indicative of statistical significance. Prior to correlation analyses, the Shapiro–Wilk test was conducted to evaluate the normality of the distributions of the SUVmean, age, SMI and average HU. Given the nonnormal distribution of some variables, Spearman's rank correlation was chosen to assess the relationships between SUVmean and other clinical parameters. To evaluate the inter‐measurer consistency of measurements, the intraclass correlation coefficient (ICC) was calculated based on assessments conducted by two independent researchers (MS and JG). Additionally, to investigate the relationship between FAP gene expression and age, we utilised the Kruskal–Wallis test, given the categorisation of age into discrete bins.

## 3 Results

### 3.1 Patient Characteristics

The patient characteristics are summarised in Table 1. There were no significant differences between the FAPI‐46 and FAPI‐74 groups in terms of age, BMI, muscle HU and SMI for both females and males. However, muscle SUVmean was significantly higher in the FAPI‐74 group compared to the FAPI‐46 group (*p* < 0.001).

**TABLE 1:** Patient characteristics.

	FAPI‐46	FAPI‐74	*p*
Age	66.6 ± 8.0	68.3 ± 10.6	0.468
Gender	F:M = 10:17	F:M = 13:14	0.493
BMI	24.5 ± 4.0	23.7 ± 2.9	0.391
Muscle SUVmean	0.76 ± 0.11	0.97 ± 0.17	< 0.001
Muscle average HU	33.0 ± 6.5	32.4 ± 6.4	0.745
SMI			
Female	37.3 ± 6.4	37.0 ± 6.3	0.882
Male	41.9 ± 5.5	40.9 ± 5.1	0.571

### 3.2 Correlation of Skeletal Muscle FAPI Uptake With Age and Muscle Density

To ensure the reliability of measurements, intermeasurer consistency was evaluated, revealing excellent ICC values of 1.00 (95% CI, 0.98–1.00) for SUVmean, 0.99 (95% CI, 0.96–1.00) for average HU and 0.96 (95% CI, 0.92–0.99) for SMI (Figure S2).

**FIGURE 1:**
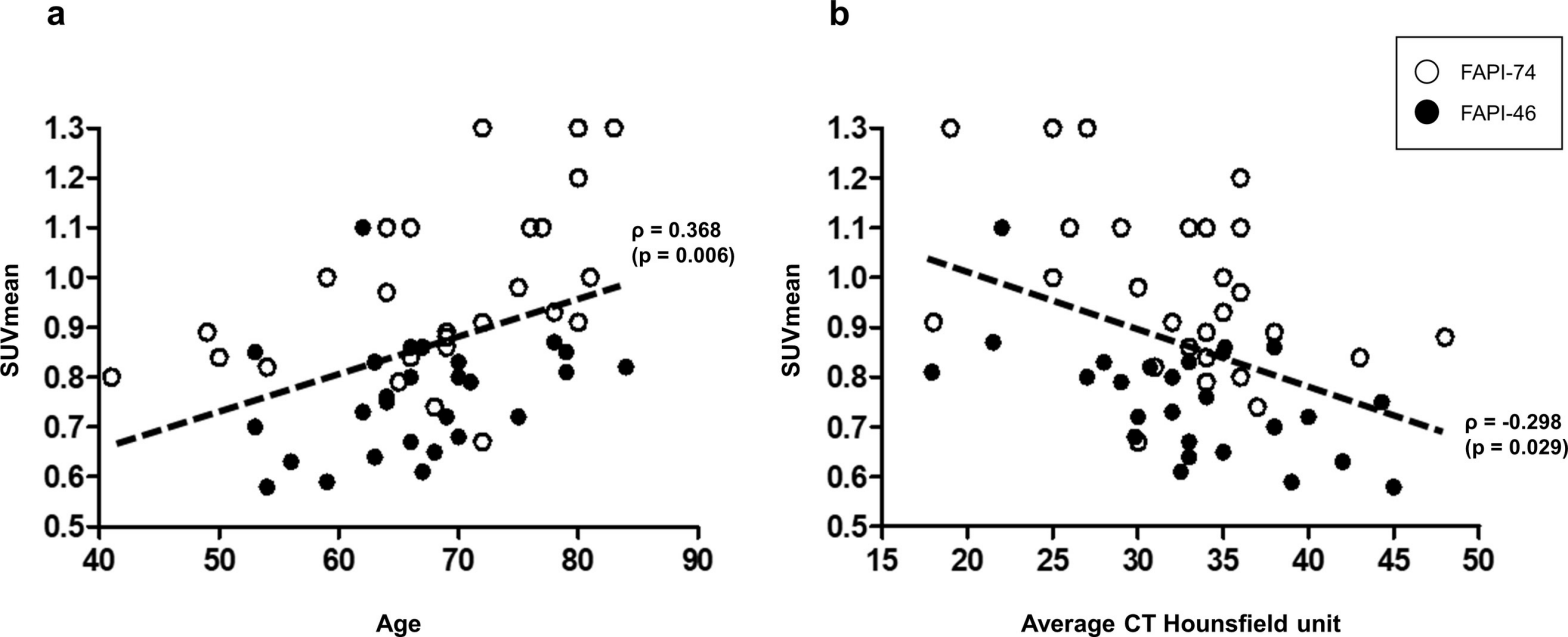
(a) Correlation between skeletal muscle SUVmean and age. (b) Correlation between SUVmean and average CT Hounsfield unit of the skeletal muscle.

The correlation analysis revealed moderate and statistically significant positive correlation between SUVmean and patient age (*ρ* = 0.368, *p* = 0.006), suggesting an age‐related increase in FAPI uptake in skeletal muscle (Figure 1A). Within the agent‐specific subgroups, the FAPI‐74 group demonstrated a strong positive correlation (*ρ* = 0.500, *p* = 0.008), indicating statistical significance. In contrast, the FAPI‐46 group showed a moderate correlation (*ρ* = 0.319), which was not statistically significant (*p* = 0.105) but indicated a trend toward increased FAPI uptake with age. Additionally, SUVmean showed a mild negative correlation with muscle average HU (*ρ* = −0.298, *p* = 0.029), indicating that higher SUVmean values are associated with lower muscle densities (Figure 1B). This trend was consistent in both subgroups, with FAPI‐46 showing a correlation of *ρ* = −0.408 (*p* = 0.035) and FAPI‐74 showing *ρ* = −0.404 (*p* = 0.037). No significant correlation was observed between SUVmean and the SMI (*ρ* = −0.034, *p* = 0.789), and this lack of correlation remained consistent when the data were analysed separately by gender.

### 3.3 Age‐Related Trends in FAP Gene Expression

**FIGURE 2:**
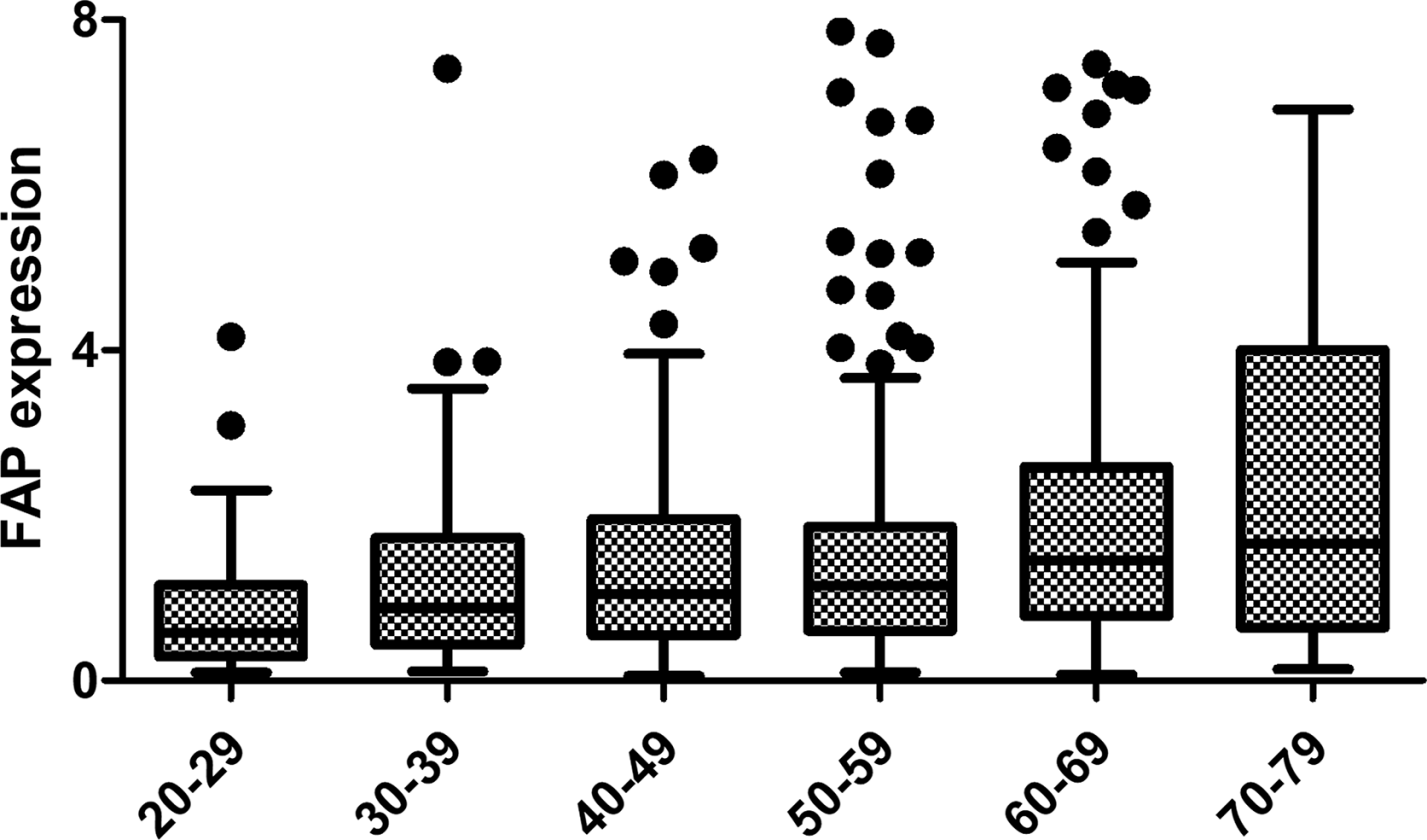
Fibroblast activation protein expression across different age groups. A box and whisker plot was generated through median, quartiles, interquartile range, and potential outliers.

The analysis revealed a significant variation in FAP gene expression across different age groups (Kruskal‐Wallis test, *p* < 0.001). Median FAP scores demonstrated a discernible increasing trend with age, with the 70–79 age group exhibiting the highest median FAP and the 20–29 age group the lowest (Figure 2). Outliers were present across all age categories, indicating substantial individual differences within each cohort.

## 4 Discussion

**FIGURE 3:**
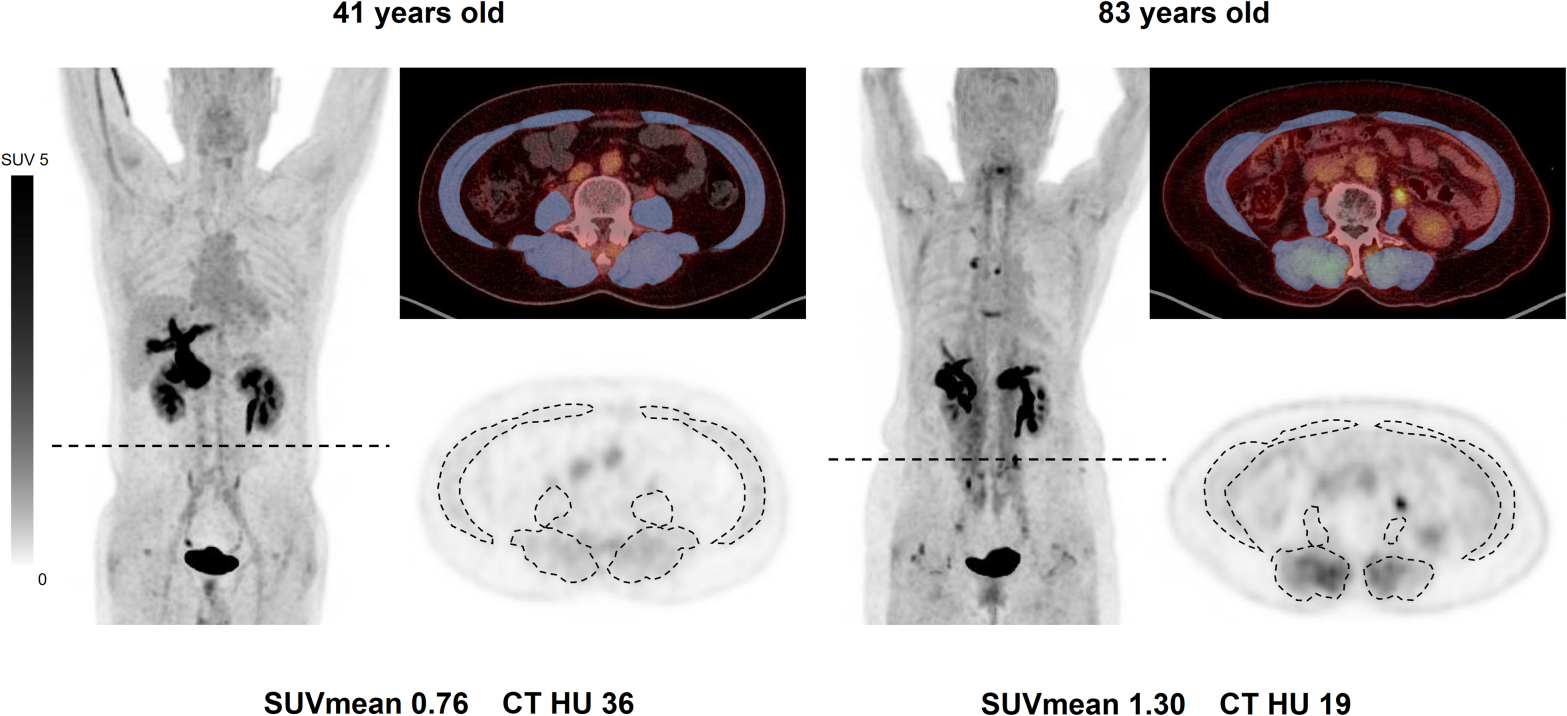
Representative image of age‐related increase in FAPI uptake in skeletal muscle.

Our study demonstrates a statistically significant, age‐related increase in FAPI uptake in skeletal muscle, with the effect being particularly pronounced in older age groups (Figure 3). A similar trend in skeletal muscle FAP gene expression was also evident in the data sourced from the GTEx. This suggests a potential biological variation in FAP expression as a function of age. Additionally, we observed a notable negative correlation between SUVmean and muscle density, indicating that higher FAP expression is associated with lower muscle densities, higher fat infiltration.

FAP is expressed in various normal tissues including adipose tissue, skeletal muscle and the pancreas, but its functions in these tissues are not well‐understood [1]. As muscles age, they often undergo increased fibrosis, where muscle fibres are replaced by fibrous connective tissue [10]. FAP expression in activated fibroblasts may drive the fibrosis and impaired regeneration in ageing muscle [11]. Furthermore, fat infiltration into muscles often occurs as a response to chronic injury or disease, where fibroblasts transdifferentiate into adipocytes [12]. The interaction between muscle fibrosis and adipogenesis can significantly worsen muscle dysfunction since both processes undermine the integrity and functionality of muscle tissue [13]. Given these roles, FAP‐expressing activated fibroblasts may play a significant role in age related muscle degeneration and fat infiltration.

On contrast, FAP may have homeostatic functions in the skeletal muscle. FAP expressing cells of skeletal muscle are known to be the major local source of follistatin, which is essential for maintaining muscle mass by inhibiting myostatin and activin A signalling [14]. The loss of these FAP expressing cells, whether through experimental ablation or pathological conditions, leads to decreased muscle mass [15]. Thus, the age‐related increase in FAP expression could potentially be an adaptive response to counteract the loss of muscle mass and strength that typically accompanies ageing. Considering the multifaceted roles of FAP in muscle tissue, where it is linked to both pathological and potentially protective mechanisms, more comprehensive research is necessary to fully understand the intricacies of its involvement in skeletal muscle.

In this study, FAPI uptake showed a relationship with muscle fat infiltration, but was not correlated with muscle mass as determined by SMI. This suggests that FAPI uptake is more closely associated with muscle quality than physical mass. Further analysis revealed that skeletal muscle FAPI SUVmean was significantly correlated with fat SUVmean in both visceral fat and subcutaneous fat regions (Figure S3). Interestingly, the observed association between skeletal muscle FAPI SUVmean and fat infiltration was more pronounced in the female subgroup, indicating potential gender‐specific differences in muscle‐fat interactions. In contrast, no significant correlations were observed with fat indices such as visceral fat index, subcutaneous fat index, or visceral‐to‐subcutaneous fat ratio, which reflect systemic fat mass. These findings highlight that localised fat infiltration within skeletal muscle may play a role in driving muscle FAP expression. Previous studies have shown that muscle fat infiltration, independently of sarcopenia, is linked to poorer survival outcomes in patients with various diseases [16, 17, 18]. Fat infiltration in the muscle leads to the buildup of lipid intermediates such as diacylglycerol and ceramide, causing insulin resistance, and is also associated with heightened systemic inflammation and oxidative stress [19, 20].

This underscores the potential of FAPI uptake as a biomarker for identifying patients with poor prognosis due to compromised muscle health. For example, elevated FAPI uptake could be used to identify patients at higher risk of adverse outcomes, such as those with cancer undergoing chemotherapy or major surgery, where muscle quality is a critical determinant of recovery and survival. Additionally, FAPI PET imaging may enable monitoring of muscle fat infiltration and fibrosis progression in chronic conditions like sarcopenia, metabolic syndrome, or neuromuscular diseases. Early detection of increased FAPI uptake could also facilitate the development and evaluation of targeted therapies aimed at modulating fibroblast activity to preserve muscle function and improve clinical outcomes.

Limitations of this study include a relatively small number of participants and its retrospective design, which may affect the generalizability of the findings. Still, these issues may be mitigated by corroborating our results with gene expression data from a broader database. Secondly, the use of two different imaging agents, [^68^Ga]FAPI‐46 and [^18^F]F‐FAPI‐74, and combining these cohorts might have introduced variability in the results due to their distinct pharmacokinetics and physiological uptake patterns [21]. [^68^Ga]FAPI‐46, with its faster blood clearance and renal excretion, may demonstrate lower skeletal muscle uptake compared to [^18^F]F‐FAPI‐74, which exhibits longer biological clearance and tissue retention. Notably, no statistically significant age correlation was observed with FAPI‐46, possibly due to its lower uptake and narrower age range. However, a trend toward increased uptake with age was observed in both agents, highlighting the need for further validation of these findings for each agent in larger, independent cohorts. Thirdly, our study included two distinct cancer cohorts, NSCLC and PDAC, which may have different effects on skeletal muscle health due to varying degrees of cancer‐related cachexia and metabolic alterations. However, as all patients underwent imaging during the preoperative staging phase, reflecting the initial disease stage, the impact on skeletal muscle parameters is expected to be minimal. Lastly, the study's focus solely on the L3 level, rather than evaluating the entire muscle uptake. However, the L3 level is a well‐established marker for total body muscle mass and is highly representative of overall skeletal muscle health [22].

## 5 Conclusion

The study revealed an age‐related increase in FAP expression in skeletal muscle, associated with changes in muscle density, potentially higher fat infiltration. These findings suggest that while FAP is typically linked to pathological conditions, its expression in uninjured muscle could indicate broader biological roles. The study highlights the potential of FAPI as an imaging biomarker for investigating muscle health and ageing. Future research should aim to expand on these findings with larger, more diverse cohorts to better understand the implications of FAP expression in muscle ageing.

## Ethics Statement

All procedures performed in studies were in accordance with the ethical standards of the institutional and/or national research committee and with the 1964 Helsinki declaration and its later amendments or comparable ethical standards. All included data were collected as study protocol approved by the local institutional ethics committee (approval No. H‐2101‐064‐1188 & H‐2112‐110‐1284). Written informed consent was obtained from all individual participants included in the study.

## Conflicts of Interest

The authors declare no conflicts of interest.

## Supporting information


**Figure S1** Skeletal muscle segmentation was performed at the L3 lumbar spine level using MIM software (MIM Software Inc., Cleveland, OH, USA). In the first step, a rough region of interest (ROI) was manually outlined on axial CT slices to include visible skeletal muscle groups, such as the psoas, erector spinae, rectus abdominis and obliques. In the second step, segmentation was refined by applying Hounsfield Unit (HU) thresholds:voxels with HU values greater than or equal to −29 were selected, and voxels with HU values greater than or equal to 150 were defined separately. In the third step, the regions with HU values ≥ 150 were subtracted from those with HU values ≥ − 29 to isolate skeletal muscle tissue with HU values ranging from −29 to 150. After segmentation, the software automatically calculated the average HU, SUVmean and muscle volume within the defined ROI. The skeletal muscle area was then derived by dividing the segmented muscle volume by the slice thickness, providing an accurate two‐dimensional measurement. ROI, region of interest; HU, Hounsfield Unit.
**Figure S2.** Scatter plots demonstrate the inter‐measurer consistency for (a) muscle SUVmean, (b) muscle average Hounsfield unit and (c) skeletal muscle index. The dotted lines represent the line of identity. HU, Hounsfield unit; SMI, skeletal muscle index.
**Figure S3.** Correlation heatmaps of skeletal muscle FAPI SUVmean and related parameters in the total cohort, male subgroup and female subgroup. Fat tissue was segmented at the L3 vertebral level using a Hounsfield Unit (HU) threshold of −190 to −30. From these regions, fat SUVmean was measured, and fat indices, including the visceral fat index (VFI) and subcutaneous fat index (SFI), were calculated as the cross‐sectional area of fat tissue (cm^2^) divided by the square of the patient’s height (m^2^). (a) In the total cohort, skeletal muscle FAPI SUVmean shows significant positive correlations with fat SUVmean in both visceral and subcutaneous regions (*ρ* = 0.67 and 0.65, respectively, *p* < 0.01). (b) In the male subgroup, no significant correlations are observed between skeletal muscle FAPI SUVmean and fat indices or fat SUVmean. (c) In the female subgroup, skeletal muscle FAPI SUVmean demonstrates stronger correlations with visceral and subcutaneous fat SUVmean (*ρ* = 0.74 and 0.75, respectively, *p* < 0.01), while also showing a notable negative correlation with muscle density (Muscle_CT_HU, *ρ* = −0.42, *p* < 0.05). HU, Hounsfield Unit; VFI, visceral fat index; SFI, subcutaneous fat index; V/S, visceral to subcutaneous fat ratio.
