# Supplementary material for: Aged‐Related Fibroblast Activation Protein Expression in Skeletal Muscles Evaluated by PET Imaging
Source: J Cachexia Sarcopenia Muscle. 2025 Feb 16;16(1):e13730. doi: 10.1002/jcsm.13730 (PMC11830633; doi:10.1002/jcsm.13730)
Supplement: Supplementary file 1 — Figure S1 Skeletal muscle segmentation was performed at the L3 lumbar spine level using MIM software (MIM Software Inc., Cleveland, OH, USA). In the first step, a rough region of interest (ROI) was manually outlined on axial CT slices to include visible skeletal muscle groups, such as the psoas, erector spinae, rectus abdominis and obliques. In the second step, segmentation was refined by applying Hounsfield Unit (HU) thresholds:voxels with HU values greater than or equal to −29 were selected, and voxels with HU values greater than or equal to 150 were defined separately. In the third step, the regions with HU values ≥ 150 were subtracted from those with HU values ≥ − 29 to isolate skeletal muscle tissue with HU values ranging from −29 to 150. After segmentation, the software automatically calculated the average HU, SUVmean and muscle volume within the defined ROI. The skeletal muscle area was then derived by dividing the segmented muscle volume by the slice thickness, providing an accurate two‐dimensional measurement. ROI, region of interest; HU, Hounsfield Unit. Figure S2. Scatter plots demonstrate the inter‐measurer consistency for (a) muscle SUVmean, (b) muscle average Hounsfield unit and (c) skeletal muscle index. The dotted lines represent the line of identity. HU, Hounsfield unit; SMI, skeletal muscle index. Figure S3. Correlation heatmaps of skeletal muscle FAPI SUVmean and related parameters in the total cohort, male subgroup and female subgroup. Fat tissue was segmented at the L3 vertebral level using a Hounsfield Unit (HU) threshold of −190 to −30. From these regions, fat SUVmean was measured, and fat indices, including the visceral fat index (VFI) and subcutaneous fat index (SFI), were calculated as the cross‐sectional area of fat tissue (cm2) divided by the square of the patient’s height (m2). (a) In the total cohort, skeletal muscle FAPI SUVmean shows significant positive correlations with fat SUVmean in both visceral and subcutaneous regions (ρ = 0.67 [file JCSM-16-e13730-s001.docx]

**Supplementary Figure 1.**

**
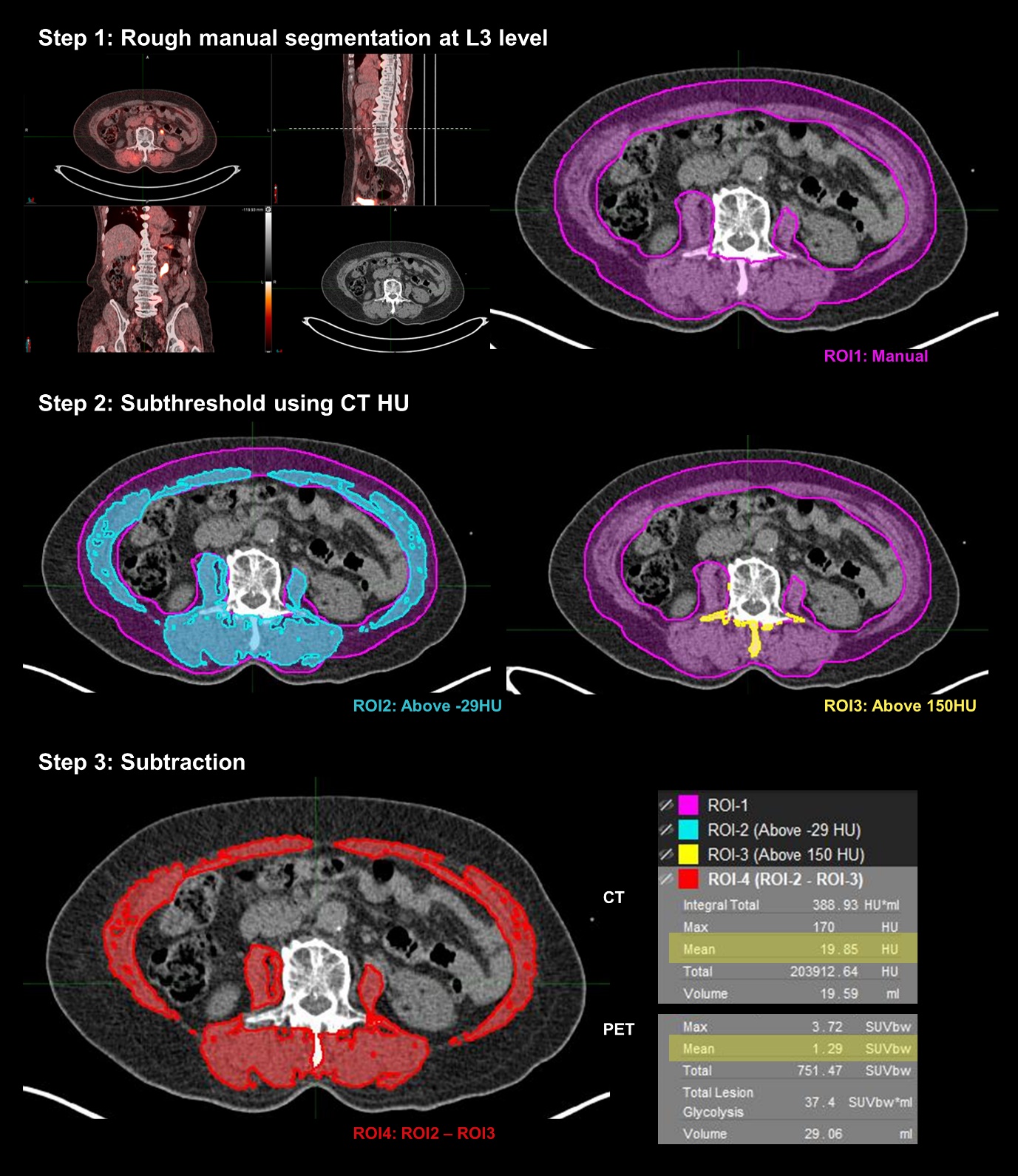
**

Skeletal muscle segmentation was performed at the L3 lumbar spine level using MIM software (MIM Software Inc., Cleveland, OH, USA). In the first step, a rough region of interest (ROI) was manually outlined on axial CT slices to include visible skeletal muscle groups, such as the psoas, erector spinae, rectus abdominis, and obliques. In the second step, segmentation was refined by applying Hounsfield Unit (HU) thresholds: voxels with HU values greater than or equal to -29 were selected, and voxels with HU values greater than or equal to 150 were defined separately. In the third step, the regions with HU values ≥ 150 were subtracted from those with HU values ≥ -29 to isolate skeletal muscle tissue with HU values ranging from -29 to 150.

After segmentation, the software automatically calculated the average HU, SUVmean, and muscle volume within the defined ROI. The skeletal muscle area was then derived by dividing the segmented muscle volume by the slice thickness, providing an accurate two-dimensional measurement. ROI, region of interest; HU, Hounsfield Unit

**Supplementary Figure 2.**


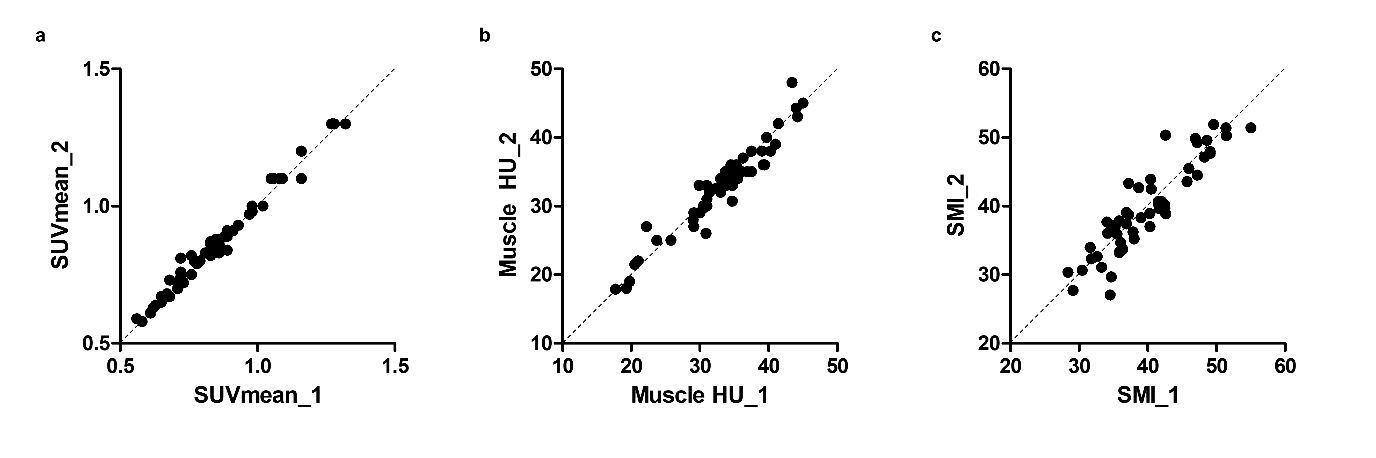


Scatter plots demonstrate the inter-measurer consistency for (a) muscle SUVmean, (b) muscle average Hounsfield unit, and (c) skeletal muscle index. The dotted lines represent the line of identity. HU, Hounsfield unit; SMI, skeletal muscle index

**Supplementary Figure 3.**

**
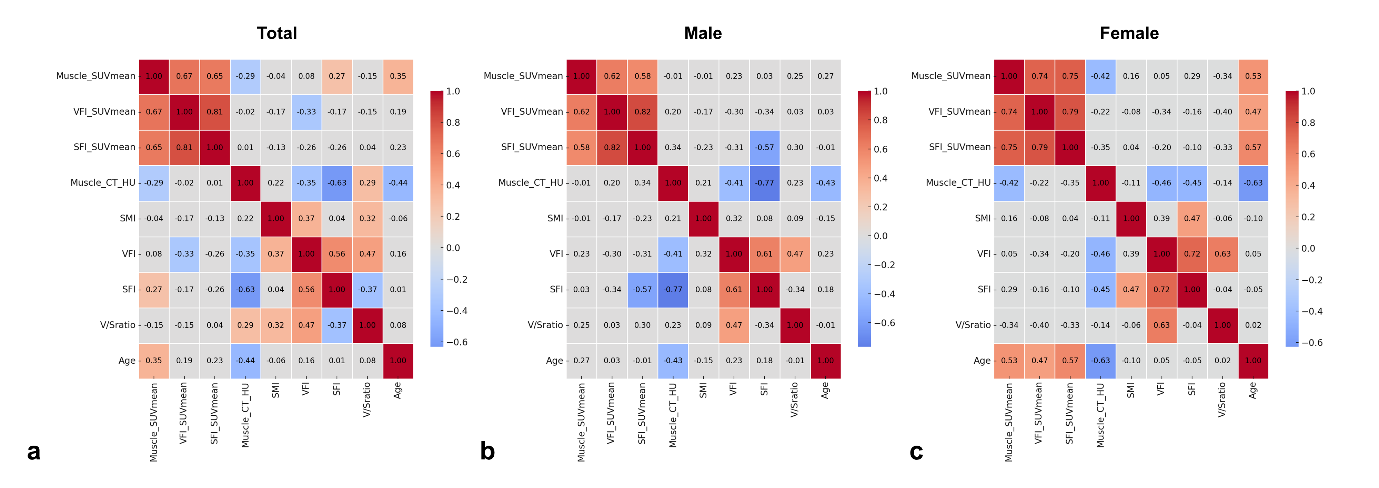
**

Correlation heatmaps of skeletal muscle FAPI SUVmean and related parameters in the total cohort, male subgroup, and female subgroup. Fat tissue was segmented at the L3 vertebral level using a Hounsfield Unit (HU) threshold of -190 to -30. From these regions, fat SUVmean was measured, and fat indices, including the visceral fat index (VFI) and subcutaneous fat index (SFI), were calculated as the cross-sectional area of fat tissue (cm²) divided by the square of the patient's height (m²). (a) In the total cohort, skeletal muscle FAPI SUVmean shows significant positive correlations with fat SUVmean in both visceral and subcutaneous regions (ρ = 0.67 and 0.65, respectively, p < 0.01). (b) In the male subgroup, no significant correlations are observed between skeletal muscle FAPI SUVmean and fat indices or fat SUVmean. (c) In the female subgroup, skeletal muscle FAPI SUVmean demonstrates stronger correlations with visceral and subcutaneous fat SUVmean (ρ = 0.74 and 0.75, respectively, p < 0.01), while also showing a notable negative correlation with muscle density (Muscle_CT_HU, ρ = -0.42, p < 0.05). HU, Hounsfield Unit; VFI, visceral fat index; SFI, subcutaneous fat index; V/S, visceral to subcutaneous fat ratio
